# A Population Survey in Italy Based on the ICF Classification: Recognizing Persons with Severe Disability

**DOI:** 10.1100/2012/189097

**Published:** 2012-02-01

**Authors:** Matilde Leonardi, Andrea Martinuzzi, Paolo Meucci, Marina Sala, Emanuela Russo, Mara Buffoni, Alberto Raggi

**Affiliations:** ^1^Neurology, Public Health and Disability Unit-Scientific Directorate, Neurological Institute C. Besta IRCCS Foundation, Via Celoria 11, 20133 Milano, Italy; ^2^IRCCS E. Medea Scientific Institute, Conegliano-Pieve di Soligo Research Centre, Via Costa Alta 37, 31015 Conegliano, Italy

## Abstract

Aim of this paper is to describe functioning of subjects with “severe disability” collected with a protocol based on the International Classification of Functioning, Disability, and Health. It included sections on body functions and structures (BF and BS), activities and participation (A&P), and environmental factors (EF). In A&P, performance without personal support (WPS) was added to standard capacity and performance. Persons with severe disability were those reporting a number of very severe/complete problems in BF or in A&P-capacity superior to mean + 1SD. Correlations between BF and A&P and differences between capacity, performance-WPS, and performance were assessed with Spearman's coefficient. Out of 1051, 200 subjects were considered as severely disabled. Mild to moderate correlations between BF and A&P were reported (between 0.148 and 0.394 when the full range of impairments/limitations was taken into account; between 0.198 and 0.285 when only the severe impairments/limitations were taken into account); performance-WPS was less similar to performance than to capacity. Our approach enabled identifying subjects with “severe disability” and separating the effect of personal support from that of devices, policies, and service provision.

## 1. Introduction

Epidemiological data on disability prevalence cannot be fully compared across countries, as some define disability in terms of performance levels in employment or other social activities while others define it in medical terms [1]. Disability population surveys generally focus and collect data, on a limited number of functional domains (e.g., activities of daily living) and on few impairments, making it difficult to identify emerging populations of persons with disability: in turn this makes it impossible to recognise features and needs of persons with problems in functioning. The connection between this problem and the eligibility criteria and benefit provision, which are a issue of policy decision making, is on the contrary less clear. In fact, responding to the needs of persons with disability is a responsibility of policymakers that need to rely on valid information to take appropriate decisions. At the same time, however, it cannot be ignored that the allocation of provisions is widely based on the availability of funds. For this reason, we deem that the achievement of an “evidence-informed policymaking,” has to be a joint effort between researchers that want their research to make a difference and policymakers, that need to use research evidence to improve the quality and effectiveness of their actions [2]. 

Diagnostic data alone do not predict health service needs, receipt of disability benefits, work performance, or social integration. What is needed are data about the full, lived experience of health, which includes functioning and disability. The International Classification of Functioning, Disability, and Health (ICF) [3] was released by the World Health Organization as the framework for documenting human functioning and disability, intended as the interaction between health condition and environmental factors, across health condition, age groups, or over time. 

In Italy, the receipt of disability benefit is subject to an evaluation of two different parameters according to persons' age. For persons aged 18–65 the requirement is the “reduction or loss of working capacity” [4], whose evaluation takes two steps: an evaluation of persisting impairments (i.e., at the level of the body) [5] and an evaluation of the limitation in working capacity due to these impairments. For persons below 18 and over 65, the requirement is expressed in terms of “persisting difficulty in undertaking age-specific tasks” [4]. The evaluation in this case takes three steps: first, an evaluation of persisting impairments (i.e., at the level of the body) [5]; second, the determination of age-specific tasks; third, an evaluation of the difficulties in undertaking these tasks due to the presence of persisting impairments. The framework on which this evaluation stems is WHO's International Classification of Impairments, Disabilities and Handicaps (ICIDH) [6], which is a predecessor of the ICF and recognises the presence of impairments, distinguishing them from disability and handicap, that is defined as the difficulty in fulfilling expected social roles. This conceptualisation determines a lack of consideration of environment's effects, which leads to a poor recognition of the needs that different persons may have in their actual context. This poor recognition determines the impossibility to predict the level of activity limitations and participation restrictions that persons with disability experience, which is a function of both bodily impairments and of environmental facilitators or barriers. Recognising the effect of person-environment interaction is essential to define the level of functioning and disability and to evaluate which situations are the most problematic or severe. 

The Italian Centre for Disease Control (Centro Controllo Malattie—CCM) funded a project aimed to define a common framework and a new protocol for disability evaluation in the Italian welfare system, based on the ICF. In the Italian welfare system the notion of disability is present in many laws, but it does not match a clear definition of assessment, and this heterogeneity increases the risk of inequity [7]. In particular, when the severity of person's disability is the focus of attention, the lack of a clear conceptual and practical framework becomes a problematic issue. 

This CCM-funded project moved from the definition of “person with disability” endorsed by the United Nations Convention on the Rights of Persons with Disabilities (UNC) [8] which highlights the effect of environmental factors, but fails in acknowledging that disability is a central health issue playing out in all life areas of individuals. Disability is in fact a state of decreased functioning associated with a health condition which in the context of one's environment is experienced as an impairment, activity limitation or participation restriction [9]: understanding both the health and environmental components of disability makes it possible to evaluate the effect of interventions that improve functioning. Such a definition of disability recognizes that disability is the outcome of the relationship between the health component, or the inner health state of the individual, and the environmental component. Therefore disability results as the negative side of this relationship, which may be due to a bad health state or to a bad environment: likely, it will be the result of both them.

Aim of this paper is to provide a representation of functional features of subjects that can be defined as “severely disabled” in terms of both impairments in body functions and limitations in executing task or activities. For the scopes of this paper, we intended for “persons with severe disability” those that report a high number of severe problems, both with body functions and with the execution of tasks or activities, thus avoiding to rely on predefined categories (e.g., wheelchair users). The definition of severe disability endorsed in this paper is purely operational and, in a sense, relative: persons are recognised as “severely disabled” compared to those that have a better functioning. Such an operational definition is however strongly grounded on a theoretical one: severe disability is a condition resulting when the relationship between the health and the environmental components is particularly unfair. Such a situation is, basically, due to the presence of a wide number of severe impairments at the level of the body, which in turn determine a high number of severe limitations in performing daily activities and in participating to social situation, and that happen in a context that might be either hindering or “not facilitating enough”, or to a further situation in which there is no environmental factor that might overcome the impairment and the limitations in executing activities due to the health condition.

## 2. Methods

### 2.1. Instruments and Procedure

This CCM-funded project involved disability evaluation committees of eight Italian regions, mostly from northern Italy. Subjects that, according to Italian legislation, presented a request to get a disability certification or undertook a periodical revision during 2009 were consecutively asked to enter in the study on a voluntary basis. Participating to the study did not affect the standard process of evaluation: it was a separate process which required to attend an one-hour evaluation, conducted by a multiprofessional team that included medical doctors, psychologists, and social workers. No age limitation or any other specific exclusion criteria was defined other than accepting to participate and signing an informed consent form: in case of children, or of adults unable to give their consent, the consent form was signed by the legal representative. Data were stored in anonymized electronic databases.

An experimental evaluation protocol was defined [7] and organised in different sections intended to collect demographic and clinical information, information on services and support currently provided, and current medical prescriptions. The core part of the protocol is composed of a section to collect information on body functions and body structures (BF and BS), and a section to collect information on activities and participation (A&P) in association with environmental factors (EF). The entire list of second-level BF and BS codes (e.g., b140-Attention functions; s310-Structure of nose) derived from the ICF-CY classification [10] was used, and each category is rated in terms of presence and severity of impairments. For the purpose of this study, we relied on the analysis of BF only. The rationale for this is that impairments at the level of the body structure might be connected to several problems with body functions, in general with a causal relationship. Since we wanted to limit the identification of causality, we restricted the analysis of bodily impairments to BF only, which usually correspond to the symptoms reported by subjects.

With regard to A&P, 70 categories were mapped to the UNC [7, 8]. Two different performance qualifiers were used. The first indicates the standard performance as defined by the ICF: the ability of a subject to execute an activity in his current environment, therefore including all available EF. The second performance index rates the ability of the subject to execute an activity with the exclusion of personal support (performance without personal support-WPS), but including assistive products, drugs, physical environment factors, and policies' effects. Finally, capacity qualifier describes the individual's intrinsic ability to execute a task or an action, therefore without EF. 

For the purposes of this project, the standardised five-point rating scale of ICF qualifiers has been modified, and only four qualifiers were available: qualifier 0, no problem (rating percentage 0–4%); qualifier 1, mild problem (problem percentage 5–24%); qualifier 2, moderate/severe problem (problem percentage 25–74%); qualifier 3, complete/very severe problem (problem percentage 75–100%). Standard qualifiers 8 and 9 (not specified and not applicable) were maintained.

The section of the protocol related to BF and BS was filled in on the basis of available medical documentation: in this case, the identification of the most precise qualifier was based on the utilisation of percentages, compared to the most adequate normative data, which is different for each ICF category. On the contrary, for the section of A&P and EF a semistructured interview was used to collect data. Persons were asked to report about difficulties in performing daily activities and to describe in what way environmental factors had an impact over them. In this case, persons were not asked to refer to percentages, but rather to express a judgement on how much their ability in performing the activity is limited, for example, in terms of how often they are able to perform the activity, how much residual ability do they have, and how much the health condition interferes with their ability. Interviewers participated to a four-day training event and were instructed to ask subjects for clarification and examples to minimize the possibility of over- or underestimation of their problems. Finally, for each single activity EF were directly linked to the corresponding A&P category.

### 2.2. Data Analysis

ICF categories rated with qualifier 8 (not specified) were replaced by each category's median value comprised between qualifiers 1–3. In case a category was never rated with qualifiers 1–3 (so the median results in a missing value), qualifiers 8 were converted into 1. ICF categories rated with qualifier 9 (not applicable) were converted into missing, because if a category is not applicable by definition, as a result there is no information on a problem's presence or severity. 

A count-based method, previously employed to analyze ICF-based data on functioning in clinical populations [11–15], was used, and for ICF domains of BF and A&P two indexes were developed: “extension” and “severity”. The first is the count of categories in which qualifiers 1–3 (describing the full range from mild-to-complete problems) were applied, whereas the second is the count of categories in which qualifiers 3 only (describing only the range of very severe to complete problems) were applied. Therefore, extension indexes' content corresponds to all the possible kind of impairments and limitations, from mild-to-complete ones, while severity indexes' content corresponds only to complete/severe impairments and limitations. Extension and severity indexes underwent a linear transformation to make them easily and directly comparable, by means of this procedure: count/max*100. Transformed values range between 0 and 100, with lowest values representing complete integrity of BF and complete absence of limitation or restriction in the A&P domains. Differences in the distribution of categories rated with qualifier 3 along the three qualifiers of A&P have been evaluated with paired-sample *t*-test. 

To select the profiles that represent a “severe disability”, two steps were taken. First, ICF categories from BF and A&P used with qualifiers 1–3 in less than 5% of cases were eliminated. Second, a selection threshold was set: persons were eligible to the definition of “severe disability” when the number of ICF categories rated with qualifier 3 in BF and in A&P-Capacity was superior to the observed mean, plus one standard deviation. Therefore, subjects could be considered as “severely disabled” either because they had a significantly higher number of impairments in BF, or because they reported a significantly higher number of limitations in A&P, or because they had both. 

Once the sample of subjects with severe profiles of functioning was identified, descriptive statistics on sociodemographic and diagnostic information were performed. Diagnosis was reported with the ICD-10, the International and Statistical Classification of Diseases and Related Health Problems [16], both at the specific three-digit level (e.g., M16-Coxarthrosis) and at the corresponding chapter level (e.g., Chapter XIII-Diseases of the musculoskeletal system and connective tissue).

Spearman's RHO correlation has been calculated to assess the relationships between impairment in BF and limitations in A&P, both extension and severity indexes. Paired sample *t*-test has been calculated to assess the difference between capacity, performance-WPS, and performance indexes (both extension and severity) for each domain, and the most used categories from the domain of EF (reported in at least 4% of cases) are reported. 

For all analysis, *P * value < 0.05 was used to set statistical significance, and data have been analysed using SPSS 11.0 and MS Excel.

## 3. Results

In total, 1051 subjects (51.3% males) were enrolled: 41% were aged 0–17, 28.9% were aged 18–64, and 30.1% were 65 or older. Paired sample *t*-test revealed significant differences in the distribution of qualifiers 3 between performance and Performance-WPS (mean 9.2 versus 17.0; *t* = 25.6, *P* < 0.001) and between performance-WPS and capacity (mean 17.0 versus 19.4; *t *= 15.9, *P* < 0.001).

### 3.1. Selection of Subjects with Severe Disability

After the elimination of ICF categories rated with qualifiers 1–3 in less than 5% of cases, the data set was reduced to 62 categories from BF and 53 from A&P. With regard to BF, mean score of severity index was 3.14 (sd 6.9): therefore, the selection threshold was 10.04. With regard to A&P-capacity, mean score of severity index was 20.13 (sd 21.57): therefore, the selection threshold was 41.7. In total, 107 subjects were selected based on BF and 172 based on A&P severity scores: of them, 79 were selected based on both BF and A&P. 

**Figure 1:**
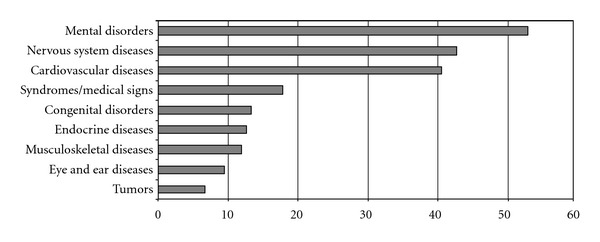
Prevalence of the most common disease group among subjects with severe disability.

Two-hundred persons (19% of the total) were included in the subsample of subject with severe disability, 58.5% were male, 47% were aged 0–17, 22% were aged 18–64, and 31% were 65 or older. Among the adults, 6.8 % declared to be employed, 2.3% were seeking for a job, and 18.2% were not employed and not looking for a job. With regard to invalidity pension, 52 subjects were at the first evaluation and therefore without a certificate: among the others, 97.3% had a certificate that recognised 100% invalidity (the percentage was 50.1% considering the whole sample). A total of 668 different diagnoses were recorded (median 3, range 1–10 per patient).  Figure 1 shows the most prevalent disease group: among mental disorders, the most common were severe mental retardation (19.4%), autism (16.7%), and Specific developmental disorders of speech and language (9%); among nervous system diseases, the most common were epilepsy (27.7%), cerebral palsy (22.7%) and para-tetraplegia (13.4%); among cardiovascular diseases, the most common were hypertensive heart disease (17.6%), cerebrovascular disease, and sequelae of cerebrovascular Disease (14.8% and 13%, resp.).

### 3.2. Profiles of Functioning of Subjects with Severe Disability

**Figure 2:**
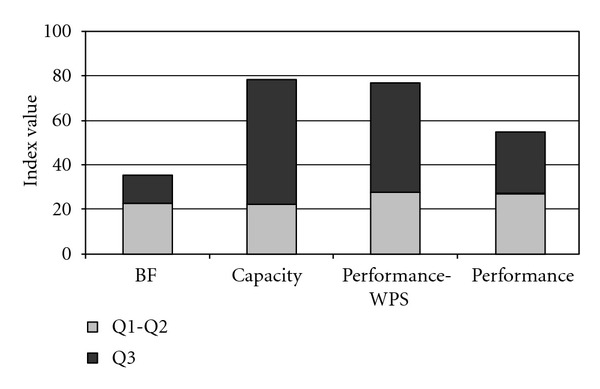
Distribution of mild-severe and very severe-complete problems among subjects with severe disability.

The mean value of extension index referred to BF was approximately half of that referred to A&P capacity and performance-WPS and was approximately 30% lower than performance index. However, as Figure 2 shows, the portion of qualifiers describing mild to severe problems (Q1 and Q2) was almost equal, and the most relevant differences were due to the portion qualifiers describing very severe/complete problems (Q3). Correlations between BF and A&P indexes are reported in Table 1. Coefficients were mild to moderate and were higher for the couples BF-capacity than for the couples BF-performance-WPS and BF-performance, and trends of significance show an association between indexes reporting similar information. In fact, extension indexes in A&P did not correlate with severity index of BF, and extension index of BF correlated only with Capacity index in A&P, and the correlations were stronger among extension indexes.

Paired sample *t-*test (Table 2) showed relevant differences between capacity and performance-WPS indexes, as well as between performance-WPS and performance indexes. It can be observed that *t-*test coefficients were higher for the pairs performance-WPS versus performance than for the pairs capacity versus Performance-WPS (with the exclusion of severity indexes referred to mobility domain). Moreover, in general *t*-test coefficients were higher when referred to pairs of severity indexes than when referred to pairs of extension indexes: exceptions refer to the domains D1-Learning and applying knowledge (capacity versus performance-WPS *t*-test was higher in extension than in severity), D7-Interpersonal interactions and relationship (capacity versus performance-WPS *t*-test was equal in extension and in severity), as well as for the domains D4-Mobility, D5-Self-care, D6-Domestic life (performance-WPS versus performance *t*-test was higher in extension than in severity).

In total, 9354 EF have been reported, (mean 46.8 per subject). The highest number of EF were used in the domains of D4-Mobility and D1-General tasks and demand (2043 and 1806, resp.). The most common EF are reported in Table 3. Thirteen have been selected in total, which constitute 76% to 92.6% of EF used in the whole subsample. The most frequently reported are e310-Support of family members, e580-Health services, systems, and policies, and e355-Support from health professionals. 

## 4. Discussion

In this paper we described the main features of subjects that could be defined as “severely disabled” enrolled in occasion of a nation-wide project aimed to assess a methodology to use ICF-related procedures for the evaluation of disability in the general population, and we provided an operational definition of severe disability. 

Less than 20% of the whole sample met the criteria we established, that is, reported a number of ICF categories from BF and A&P domains significantly higher than the average of the sample. In this group, the portion of males was slightly higher than that observed in the whole sample (58.5% versus 51.3%), and the portion of subjects aged 18–64 was lower (22% versus 28.9%). This is contrasting with the results of the Italian National Survey on Health Conditions and Access to Health Services (INS), conducted in 2004-2005 on the basis of approximately 60.000 families [17]. The two projects did not rely on similar definitions of disability, and some differences on reported data exist: the male/female ratio is profoundly different, as INS results account for a percentage of 3.3% of males and 6.1% of females which were defined as “disabled” in the INS survey, and the percentage of subjects declaring three or more chronic diseases (which corresponds to the median observed in the present study) was 10.3% for males and 17.2% for females. 

Age is one of the major driver for increased prevalence of chronic conditions and problems in functioning. The phenomenon known as “compression of morbidity” [18]—namely, the hypothesis that the burden of lifetime illness may be compressed into a shorter period before the time of death—will have a dramatic effect on health policies, as the number of old or very old subjects is increasing and expected to increase more and more worldwide. The ageing of Italian population is among the most rapid in Europe. Percentage of persons aged 60+ will rise from 24.1% to 34% between 2000 and 2025 (and will rise from 3.9% to 7.5% for those 80+), resulting in a rise in median age from 40.2 to 50.7 [19]. Our results are consistent with these epidemiological findings, as the majority of adult subjects, from both the whole sample and the subsample of persons with severe status, were aged 65+. What is interesting to notice is that the relative percentage of children and youth is higher among the subsample of subjects with severe condition than among the whole sample. This is consistent with the fact that the most prevalent disease, that were recorded (i.e., severe mental retardation, autism, developmental disorders of speech and language, epilepsy, and cerebral palsy) are usually diagnosed during childhood or adolescence. 

Data referred to disability features indicate that the relationships between the amount of impairments in BF and the amount of problems in A&P are stronger when the overall number of problems, rather than the portion of very severe and complete ones, is taken into account. In a sense, presence of severe BF impairment is connected to the presence of severe problems in A&P: such a relationship is however only mild or moderate. We hypothesize that such a relationship is connected to the features of the most prevalent diseases observed in the sample. The majority of them (e.g., cerebral palsy, para-tetraplegia, stroke and stroke sequelae) typically determine problems with both mental and movement functions that, in turn, might determine a wide range of problems with learning, communication, mobility, self-care, daily activities, employment, or school. What is also observed is that the difference between performance and performance-WPS indexes is wider than that observed between performance-WPS and capacity indexes. Basically, this means that most of the facilitating effect of environmental factors is due to the presence of persons that actively provide support to the individual. Consistently, we found that the support from family members was the most relevant and widespread facilitator across all A&P domains. The issue of the relevance of family support is known when disability issues are taken into account and was recently included within the policy recommendations of an European Commission-funded project. Support from family members resulted across all conditions and settings, both in adults and children, thus making it clear the need of reviewing disability policies to emphasize and support the role of the family [2]. However, if on one side the relevance of family support is a data-derived fact, we also have to acknowledge that a wide portion of subjects were aged 0–17, and therefore their level of support from family members is of different quality compared to persons in other age groups. 

The analysis of the distribution of EF in connection to the differences observed between A&P indexes also provides interesting cues for further analysis. First, strong differences between indexes, as measurable by *t-*test values, are connected to the presence of core EF. This is the case of the domains of self-care and domestic life, which are strongly connected to presence of support from family members. The second interesting aspect is the specificity of some of the reported EF. In fact, while the support from family members and from health-related professionals and the presence of effective health services, systems, and policies are common to almost all domains, other EF are more specific. This is the case, for example, of the support from friends that specifically targets major and social life domain, as well as of technologies for mobility that directly impact on the A&P domain of mobility itself, or of products for communication that target the domains of applying and learning knowledge and of communication. Further analysis should be devoted to understanding the role of EF in relationship to improved functioning, but this is beyond the aim of the present paper. 

The definition of disability on which we relied enabled us to project our methodology and our results in a broader perspective, where disability is not the result of an evaluation process only, but is the result of the interaction between the individual (with his health condition and the impairments and limitations/restrictions that he experiences as a consequence of it) and the contextual factors modulating his functioning. An outstanding example of the value of the information added by an ICF-based approach like the one we proposed in the present paper is the percentage of subjects reporting 100% invalidity certificate. Among the sample of subjects with severe disability, almost all the persons had this certificate, and considering the whole sample, half of the them had this certificate. The project from which these data are derived aimed to define a methodology for future development of the way in which disability is certified in Italy. It is clear that the two methods of evaluation (i.e., the standard one, based on percentages, and that presented in this paper) collect different kinds of information out method is certainly much more comprehensive and, in our opinion, provides data that are more directly transposable into an action that may be beneficial to the individual. For example, reporting that family members' support is crucial in almost all domains of activities means that there is an important burden of care and assistance over families. Considering the demographic features of the sample of subjects with severe disability, we can assume that majority of caregivers are elderly people (in case of husbands or wives of persons aged over 65), or young parents that will be in need to care for their sons and daughters for a prolonged period of time. In both these two situations, some factual support should be provided to family members to prevent possible health problems as well as to enable the continuation of a remunerative employment. For these reasons, we firmly believe that we were able to provide the elements for an “evidence-informed policymaking”.

Some limitations need to be taken into account in the interpretation of our results. The first is the cross-sectional design which does not allow us to determine causal relationships. A second limitation lies in sample composition. Sample was in fact composed of subjects (or their legal representatives) that participated in the interview on a voluntary basis, and the subjective motivations for accepting or refusing were not investigated. Third, although a comprehensive and consistent training was provided to interviewers to get the most adequate and precise information possible, the possibility of an over or underestimation of subjects' performance cannot be completely excluded. This caution is particularly relevant when persons were required to rate their performance without personal support, and more in particular in comparison to capacity, while the difference between the two indicators of performance is sharp and not ambiguous. Finally, although the total number of enrolled subjects is high, careful attention should be paid to a full generalisation of results to the Italian population of persons with disability. Sample was unbalanced between northern and central-southern regions and, within participating regions, only a restricted number of districts were covered. The result is that the majority of enrolled subjects were from urban contexts, which have different degree and quality of access to health and social services than those from a rural context. 

## 5. Conclusions

This paper presented a representation of functional features of subjects that can be defined as “severely disabled” in terms of both impairments with body functions and of their limitations in executing task or activities. The definition of disability which we relied upon is based on the ICF framework, and we proposed an operational definition of “severe disability” which is consistent with the frame of reference. 

Our data collection methodology enabled us to separate the effect of formal and informal support, from that of devices, policies, and service provision, and to highlight that the most relevant impact is due to the effect of support from others and in particular from family members. Further studies are needed to better understand the role of BF impairments and presence of EF on the amount of limitations and restrictions that persons with disability confront with.

## Figures and Tables

**Table 1:** Spearman's RHO correlation between BF and A&P indexes (*N* = 200).

Activities and participation	Body functions	
	Extension index	Severity index
Extension index		
Capacity	.394**	ns
Performance WPS	.364**	ns
Performance	.148*	ns

Severity index		
Capacity	.225**	.285**
Performance WPS	ns	.208**
Performance	ns	.198**

*P < 0.05; **P < 0.01; ns: not significant.

**Table 2:** Paired-sample *t-*test between A&P qualifiers of capacity, performance-WPS, and performance (*N* = 200).

Domain and index		Activities and participation-mean (SD)		Paired sample *t*-test
Full A&P set of categories	Extension	Capacity	78.5 (15.5)	6.0** 28.8**
		Performance-WPS	77.5 (16.0)	
		Performance	54.8 (17.0)	
			
	Severity	Capacity	55.6 (18.1)	10.8** 29.7**
		Performance-WPS	49.8 (18.7)	
		Performance	27.4 (15.2)	

D1—learning and applying knowledge	Extension	Capacity	64.5 (24.3)	4.7** 5.9**
		Performance-WPS	63.2 (24.6)	
		Performance	59.0 (26.8)	
	Severity	Capacity	42.8 (26.4)	4.6** 10.0**
		Performance-WPS	40.8 (26.8)	
		Performance	30.4 (23.0)	

D3—communication	Extension	Capacity	82.9 (26.4)	2.1* 5.6**
		Performance-WPS	82.3 (27.2)	
		Performance	78.0 (30.7)	
	Severity	Capacity	58.9 (30.9)	4.8** 10.4**
		Performance-WPS	55.8 (31.2)	
		Performance	41.1 (28.0)	

D4—mobility	Extension	Capacity	76.8 (29.9)	3.6** 13.2**
		Performance-WPS	75.6 (30.4)	
		Performance	59.6 (27.6)	
	Severity	Capacity	51.1 (33.5)	9.8** 8.0**
		Performance-WPS	38.1 (29.0)	
		Performance	27.4 (21.2)	

D5—self care	Extension	Capacity	90.5 (18.1)	2.6* 36.2**
		Performance-WPS	89.7 (18.8)	
		Performance	14.8 (25.5)	
	Severity	Capacity	62.6 (29.8)	7.6** 25.2**
		Performance-WPS	53.3 (29.0)	
		Performance	3.2 (10.3)	

D6—domestic life	Extension	Capacity	91.2 (24.8)	NA 29.1**
		Performance-WPS	91.2 (24.8)	
		Performance	32.7 (26.6)	
	Severity	Capacity	87.6 (27.5)	2.7** 26.8**
		Performance-WPS	86.2 (28.1)	
		Performance	30.0 (26.6)	

D7—interpersonal interactions and relationships	Extension	Capacity	76.3 (29.3)	1.0 7.1**
		Performance-WPS	76.5 (29.4)	
		Performance	67.6 (32.5)	
	Severity	Capacity	45.3 (35.0)	1.0 10.0**
		Performance-WPS	44.8 (35.1)	
		Performance	27.2 (28.1)	

D8&9—major and social life areas	Extension	Capacity	84.4 (25.6)	5.1** 16.9**
		Performance-WPS	81.5 (26.2)	
		Performance	51.2 (29.3)	
	Severity	Capacity	68.4 (30.7)	6.2** 17.6**
		Performance-WPS	61.6 (32.3)	
		Performance	27.9 (30.1)	

*t-*test was not executed for the pair capacity and performance-WPS in D6—domestic life because the standard error of the difference was = 0. *P < 0.05; **P < 0.01.

**Table 3:** Mean percentage of EF categories reported for each domain of A & P.

Categories from domains of environmental factors	Domains of activities and participation							
	D1 (*n* = 1806)	D3 (*n* = 1395)	D4 (*n* = 2043)	D5 (*n* = 1502)	D6 (*n* = 712)	D7 (*n* = 904)	D8 & 9 (*n* = 992)	Dtot (*n* = 9354)
e115—products and technologies for personal use	1.5	1.0	**6.9**	**4.5**	0.8	1.0	0.9	2.9
e120—products and technologies for indoor and outdoor mobility	0.0	0.0	**10.5**	1.9	1.5	0.0	1.2	2.8
e125—products and technologies for communication	**8.7**	**8.5**	0.4	0.0	0.1	0.3	0.9	3.2
e165—assets	2.8	2.9	1.4	1.7	**4.5**	1.8	**4.8**	2.6
e310—support from immediate family	**20.4**	**22.5**	**23.6**	**40.3**	**54.4**	**25.7**	**33.1**	**29.1**
e320—support from friends	0.3	0.8	1.1	0.8	1.1	3.7	**8.2**	1.9
e340—support from personal care providers and assistants	**4.7**	3.8	3.8	**6.5**	**6.9**	2.7	2.6	**4.4**
e355—support from health professionals	**11.7**	**12.9**	**13.6**	**8.4**	2.2	**8.6**	3.9	**9.9**
e360—support from health-related professionals	**13.2**	**12.7**	**4.5**	**5.7**	3.4	**8.5**	**4.5**	**7.9**
e410—individual attitudes of family members	2.8	1.8	1.8	2.5	3.1	**5.9**	2.3	2.6
e570—social security SSP	0.3	0.2	0.3	0.7	2.9	0.4	**7.4**	1.3
e575—general social support SSP	3.6	2.9	2.1	2.7	**4.9**	2.5	2.7	2.9
e580—health SSP	**11.7**	**13.1**	**16.6**	**11.7**	**4.8**	**9.8**	**6.7**	**11.8**
e585—education and training SSP	**10.9**	**8.3**	2.2	3.1	0.3	**5.1**	2.0	**5.1**

Total	92.6	91.4	88.8	90.5	90.9	76.0	81.2	88.4

For each domain, the total number of categories from EF is reported in columns' headings between brackets. Categories that were never reported at least in 4% in at least one of the domains were dropped; values higher than 4% are reported in bold. D1: General tasks and demand, D3: Communication, D4: Mobility, D5: Self-care, D6: Domestic life, D7: Personal interactions and relationships, D8 and D9: Major and social life areas, Dtot-Full A&P set of categories; SSP = Services, Systems, and Policies.
